# Characterisation of the *Escherichia coli *membrane structure and function during fedbatch cultivation

**DOI:** 10.1186/1475-2859-3-9

**Published:** 2004-07-28

**Authors:** Atefeh Shokri, Gen Larsson

**Affiliations:** 1 The Swedish Centre for Bioprocess Technology, Albanova University Centre, KTH SE-106 91 Stockholm, SWEDEN

## Abstract

**Background:**

Important parameters during recombinant protein production in *Escherichia coli, *such as productivity and protein activity, are affected by the growth rate. This includes the translocation of protein over the membrane to gain better folding capacity or reduced proteolysis. To vary the growth rate two techniques are available: fedbatch and continuous cultivation, both controlled by the ingoing feed rate.

**Results:**

During fedbatch cultivation, *E. coli *contains phosphatidylethanolamine, phosphatidylglycerol, cardiolipin and saturated fatty acids in amounts which are stable with growth rate. However, the levels of cardiolipin are very high compared to continuous cultivation. The reason for fedbatch triggering of this metabolism is not known but hypothesised to result from an additional need for carbon and energy. The reason could be the dynamic and sometimes rapid changes in growth rate to which the fedbatch cell has at all times to adjust. The membrane flexibility, essential for translocation of various components, is however to some degree sustained by production of increased amounts of unsaturated fatty acids in phosphatidylglycerol. The result is a functionally stiff membrane which generally promotes low cell lysis and is constant with respect to protein leakage to the medium. At comparatively high growth rates, when the further stabilising effect of cyclic fatty acids is gone, the high level of unsaturated fatty acids results in a pronounced effect upon sonication. This is very much in contrast to the membrane function in continuous cultivation which shows very specific characteristics as a function of growth rate.

**Conclusions:**

The stiff and unchanging fedbatch membrane should promote a stable behaviour during downstream processing and is less dependent on the time of harvest. However, optimisation of protein leakage can only be achieved in the continuously cultivated cell where leakage is twice as high compared to the constant leakage level in fedbatch. If leakage is undesired, continuous cultivation is also preferred since it can be designed to lead to the lowest values detected. Induction at low growth rate (<0.2 h^-1^) should be avoided with respect to productivity, in any system, since the specific and total protein production shows their lowest values at this point.

## Background

For several recombinant proteins produced in *Esherichia coli *there seems to be a strong dependence of the productivity and product quality on the limiting substrate feed rate at induction [1-8]. Different feed profiles in fedbatch cultivation have thus been used to affect the growth rate and thus the production of the desired product. A common strategy to increase cell productivity and product quality is furthermore to export products to the periplasm. This reduces proteolysis since the periplasm contains less proteases and the more oxidising environment is favourable for correct folding of proteins containing disulphide bridges. Furthermore, the translocation to the periplasm can result in a primary purification provided that the outer membrane can selectively be removed which requires a stable inner membrane.

Of particular importance for protein translocation processes are the anionic phospholipids but also unsaturated fatty acids. It was reported a great number of times that increased amounts of phosphatidylglycerol could facilitate protein translocation [9,13-15]. In continuous cultivation data has shown that there is clear growth rate coupling of the membrane phospholipid/fatty acid structure [9,10] but also a coupling between the structure and function [9]. It is also known that the accumulated amount of protein translocators are dependent on for example the medium composition but are also influenced by metabolic events like catabolite repression [11,12]. The latter process is known to be a function of the growth rate [12].

In continuous cultivation it was observed that conditions for establishment of an optimum in phosphatidylglycerol and unsaturated fatty acids could be achieved. An optimum in the accumulation of these compounds occurred at a growth rate of 0.3 h^-1 ^simultaneously to an optimum in *β*-lactamase leakage over the outer membrane [9]. It was further shown that at already at growth rates lower than 0.3 h^-1 ^unsaturated fatty acids where replaced by cyclic fatty acids [9] which was earlier thought to take place first in stationary phase, as shown in batch cultivation experiments [26,27]. If the formation of cyclic fatty acids is allowed to take place this influences some important functions of the membrane due to the higher rigidity caused by this rapid accumulation. This is of great concern since this generally constitutes the point of induction in protein producing processes but also since the cell organelles have a particular influence on the unit operations of the cell harvest and further in the downstream processing performance.

There is thus a strong incentive to select and operate processes which allow the establishment of controlled growth conditions and to understand the specific growth characterises established with respect to the membrane at particular feed rates. Two main techniques are at present available: fedbatch and continuous cultivation. We have previously established data from continuous cultivation [9] which we here will compare to the present data from fedbatch cultivation.

The fedbatch technique is used when a high cell density is the primary goal. A variety of feeding strategies can be applied for different research purposes where exponential feed can be used to create conditions that resemble a steady state in all points except for the continuous cell mass accumulation. This technique can however only be used for a few number of generations since the oxygen transfer rate rapidly becomes limiting. In order to reach a high cell density, i.e. cell densities of 50 g, l^-1 ^and above, the traditional fedbatch concept established early in the former century based on bakers yeast and later on for antibiotics production, is generally used. The feed profile, characterised by this general concept, is initially exponential until the oxygen or heat transfer capacity of the reactor is reached and thereafter constant. This implies that the substrate concentration and the specific growth rate decline with cultivation time after the constant feed profile is adopted. This constitutes the advantage: the declining growth rate allows the cell mass productivity to increase at constant oxygen consumption/heat production. A further advantage of this cultivation mode, in comparison to a standard batch process with substrate overflow and to fedbatch with high exponential feed, is the possibility to avoid inhibitory by-products such as acetate. At a preset time during the feeding process, induction is performed. The drawback is that when the cell mass is very high the substrate consumption is very low and this point might not represent the optimal condition for high product formation, achievement of high product quality or facilitated transport of the protein.

In continuous cultivation the feed/dilution rate is also used to control the growth rate where there is a limit of the chemostat to cultivation at the maximum or near maximum rates when the pH-auxostat is the preferred method [16]. Continuous cultivation relies on a theoretical steady state in all variables and the production can thus take place under constant growth conditions. Fedbatch cultivation for high cell density production, on the other hand, is a dynamic operation, as described above, where the cell at all times has to adapt to a new environment. Although the processes can be designed for induction at the same growth rate, the overall cell response might still severely differ due to the difference in process conditions preceding the induction, sometimes referred to as a result of the microbial "memory" of the recent past. One reason is that the time of adaptation of important cellular processes related to production and product quality by far exceeds those of the changes in substrate uptake. Thus, after a rapid change in the feed rate it might very well take some time before seen in a product variable. In spite of all benefits and drawbacks characterising both techniques, the fedbatch technique, using the concept described above, is the most common production technique in industry for recombinant production of protein pharmaceuticals in *Escherichia coli*.

The goal of this work is to show how growth rate controls the performance in fedbatch cultivation with respect to the structure of the membrane and to compare these changes to the function of some selected process variables and methods. These data will, in turn, be compared to earlier data derived from continuous cultivation [9]. From this, conclusions could be drawn as to which are the bottlenecks of either method at a particular event which will facilitate the choice of production method and enhance the design of the growth rate profile of the chosen method to achieve an increased cultivation but also harvest and downstream processing performance.

## Results

**Figure 1:**
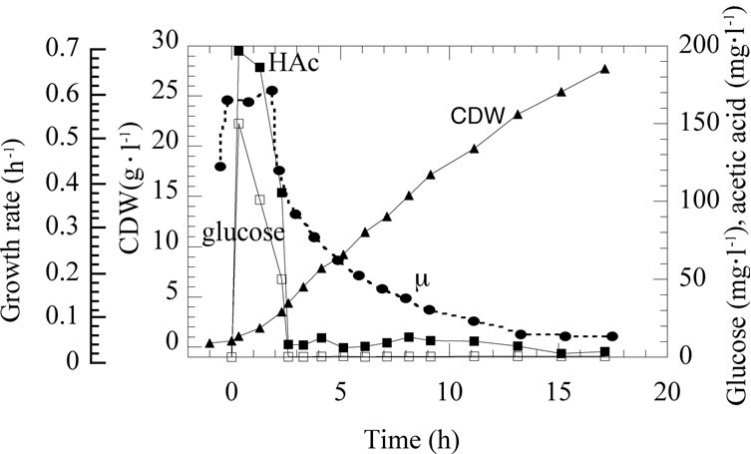
Time course of the fed-batch cultivation with *E.coli *W 3110. The process is started at time zero with an exponential feed phase corresponding to a specific growth rate of 0.6 h^-1^, followed by constant feed at approximately 2,5 h. Filled circles: specific growth rate (h^-1^), filled squares: acetic acid accumulation (mg, l^-1^), triangles: cell dry weight (CDW,g, l^-1^) and open squares: glucose accumulation (mg, l^-1^).

*Escherichia coli *W3110, a K-12 derived strain, was grown in fedbatch cultivation. The fedbatch concept was characterised by an initial exponential feed, started at time zero, of the growth limiting component, glucose, leading to the establishment of a specific growth rate of 0.6 h^-1^. This was followed by a constant feed phase where the growth rate declined asymptotically to a value of approximately 0.05 h^-1^. During this phase the cell mass increases linearly to a cell density of approximately 27 g, l^-1^, as expected (Figure 1). The characteristic and rapid declination in the growth rate, following the shift from exponential to constant feed, is also shown in the figure where the growth rate thus decreases by half in a little more than two hours. This feed process was designed to cover the larger part of the possible growth rates achievable with this strain of *E. coli *to permit comparison with earlier data from continuous cultivation [9]. However, the profile follows the general industrial feed profile concept except for the levels, which are generally kept below the limit for acetic acid production [9]. During the exponential feed phase acetic acid is thus formed however only in minor amounts due to the high glucose influx which induces overflow metabolism. This amount of acetic acid is consumed concomitantly to glucose during the rapid declination of the growth rate from 0.6 h^-1 ^to 0.4 h^-1^.

**Figure 2:**
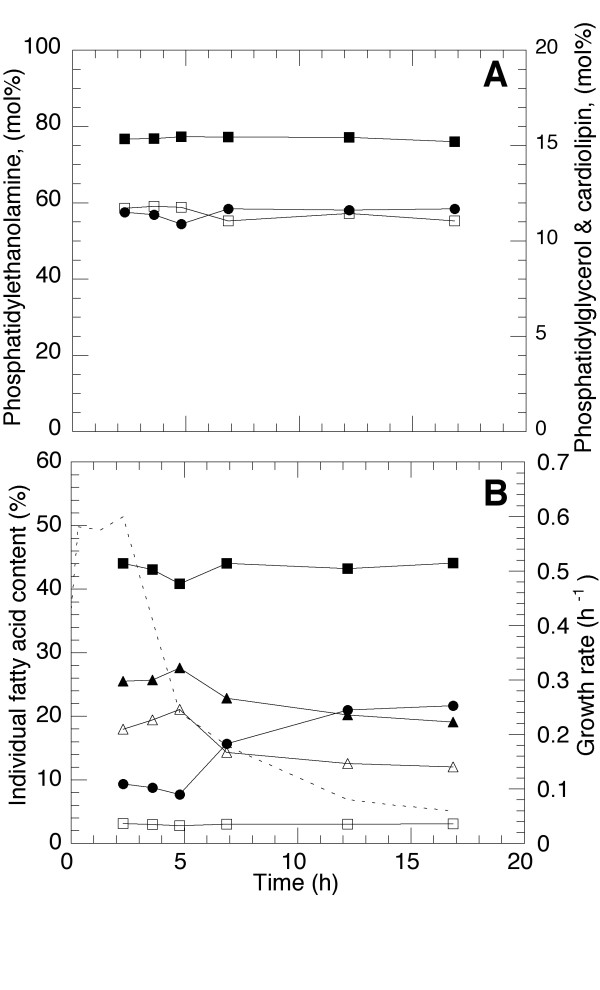
A. Accumulation of phospholipids as a function of cultivation time. Filled squares: phosphatidylethanolamine (PE), open squares: phosphatidylglycerol (PG), circles: cardiolipin (CL). B. Accumulation of fatty acids as a function of time. Open squares: C14:0, filled squares: C16:0, filled triangles: C16:1, open triangles: C18:1, circles: C17cyc. The specific growth rate is indicated in the figure by a dashed line.

The phospholipid content of the *E.coli *membranes is shown as a function of time from constant feed start in Figure 2A. The amount of phosphatidylethanolamine (PE) is always predominant and constitutes 76–77% of the phospholipids in the cell membranes at all growth rates. The content of phosphatidylglycerol (PG) and cardiolipin (CL) is kept at a level slightly below 12 % throughout the cultivation and does not vary to a great extent. At a growth rate of approximately 0.3 h^-1 ^a minimum of CL of 10.9 % is indicated which is also the time of the maximum PG level. At lower growth rates there is less PG than CL which is directly opposite to the amounts of these compounds at high growth rates. Apart from these small changes the phospholipid values are constant throughout the cultivation.

The fatty acids are shown as a function of the time from constant feed start in Figure 2B. The fatty acids predominant in the *E.coli *cytoplasmic and inner part of the outer membrane, are saturated (SFA: C14:0, C16:0), unsaturated (UFA: C16:1, C18:1), and cyclic (CFA: C17:cyc). From this figure is evident that the amount of SFA's is the most stable during the declination of growth. There is only a slight reduction of the amount at a growth rate of approximately 0.3 h^-1^, which is accompanied by a maximum in the amount of unsaturated fatty acids. The unsaturated fatty acids are markedly decreased as the growth rate goes below 0.3 h^-1 ^to 56/70 % of their maximum values, respectively. These maximal values, which coincide with the above mentioned minimum in CL/maximum in PG, thus constitutes a switch-point in the fatty acid and phospholipid metabolism during fedbatch cultivation. At this point there is also a minimum of cyclic fatty acids. The levels of the cyclic fatty acids are then rapidly increasing the lower the growth rate and from its minimum the cyclic fatty acids are dramatically increased by almost 100 % in less than two hours. This occurs although there is a supply of nutrients at all times which is in contrast to earlier literature where it is stated that it is the lack of carbon and energy in the stationary phase which results in cyclic fatty acid formation. During the continuing declination of the growth rate, the cyclic fatty acids continues to increase to a final of three times their minimum value. It should be noted that since the fatty acids of the lipopolysaccharide (LPS) were removed during the sample extraction, the results are only reflecting the inner part of the outer membrane and the cytoplasmic membrane.

**Figure 3:**
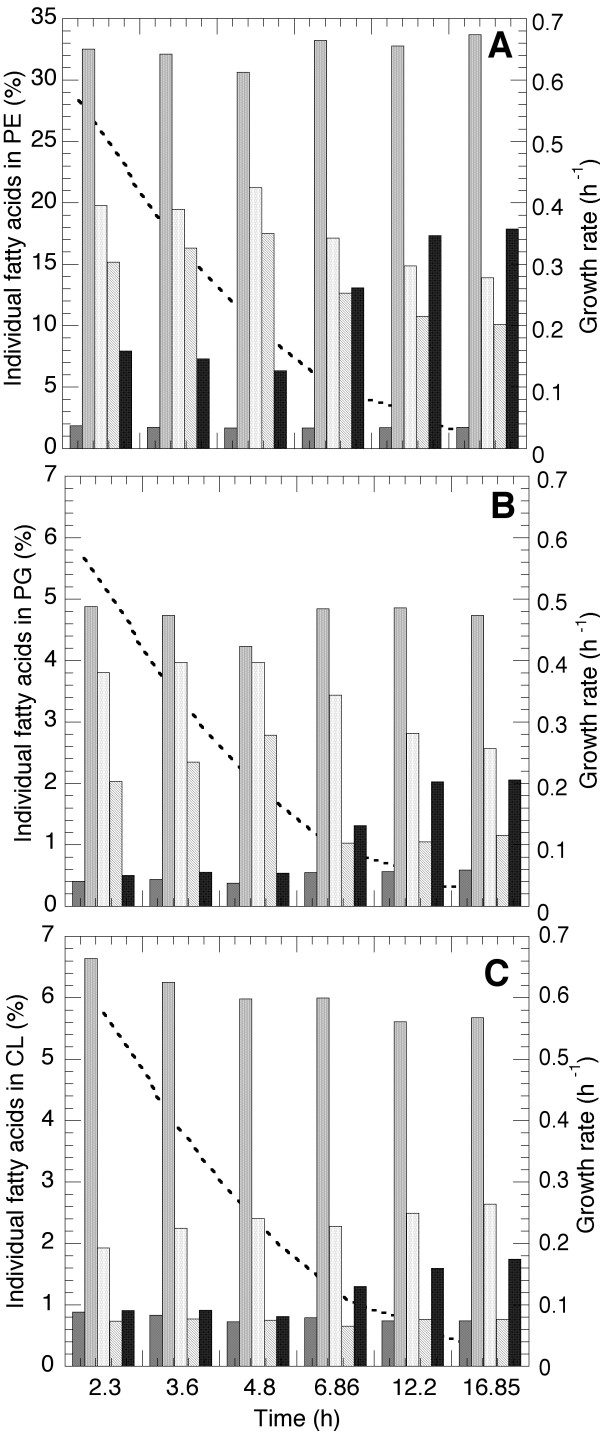
Accumulation of fatty acids in the specific phospholipids PE, PG and CL. The five bars from left to right at each time event in the figure are: C14:0, C16:0, C16:1, C18:1, C17:cyc. The specific growth rate is indicated in the figure by a dashed line.

The three phospholipids were furthermore examined for their individual fatty acid content. The results are shown in Figure 3. Since the dominating phospholipid is PE the pattern of fatty acids in PE consequently mirrors the overall results seen in Figure 2B. The amount of saturated fatty acids does not change to a high degree in any phospholipid, the differences lie more in the changes of unsaturated and cyclic fatty acids. In PG the same general pattern as in PE is observable but the increase in C18:1 with growth rate is more pronounced and this increase is made at the expense of C17:cyc. In CL, it is obvious that the levels of the fatty acids do not change very much at all except for the well-documented increase in cyclic fatty acids towards low growth rate. Since the metabolic pathway depicts that the formation of CL is a condensation of two molecules of PG and since the fatty acid pattern in CL is not mirrored by the one in PG it is thus evident that there is a modification also of the fatty acids during the condensation. This is in favour of considerably less unsaturated fatty acids in CL specifically with respect to C18:1 and also less cyclic fatty acids compared to PG. The difference is however less at lower growth rates.

The effects of these membrane changes were investigated on basis of the function of some selected methods and process variables: the mechanical strength of the membrane to ultrasound, the effect of osmotic chock, the shredding of endotoxin to the medium, the leakage to the medium and the amount of cell lysis.

**Figure 4:**
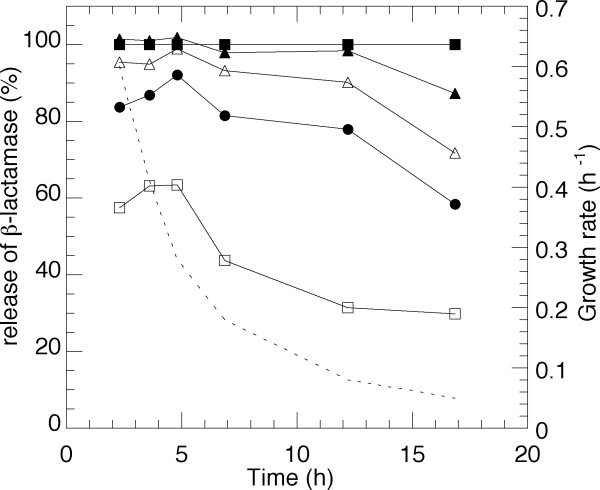
Effect of sonication time on *E. coli *W3110 shown as *β*-lactamase release compared to French press data. Top filled squares: French press data. From top to bottom: 2, 1.5, 1, 0.5 minutes sonication at constant effect and cell mass. The specific growth rate is indicated in the figure by a dashed line.

The mechanical strength of the membrane during different growth rates was quantified as the release of the periplasmic marker, *β*-lactamase, at different time of sonication using the same amount of cell and sonication effect. Figure 4 illustrates the release compared to French press data. A full release of the amount of the specific protein is achieved in all cases by a two-minute sonication except for the samples at the lowest growth rate. There is no evident change in the pronounced effect of sonication at growth rates down to 0.3 h^-1 ^but from this point on there is continuously increased membrane stability. A comparison to the pattern of unsaturated fatty acid accumulation in Figure 2B shows the close resemblance between structure and performance and where the high amounts of the double bond might be the reason to the effect of the ultrasound.

**Figure 5:**
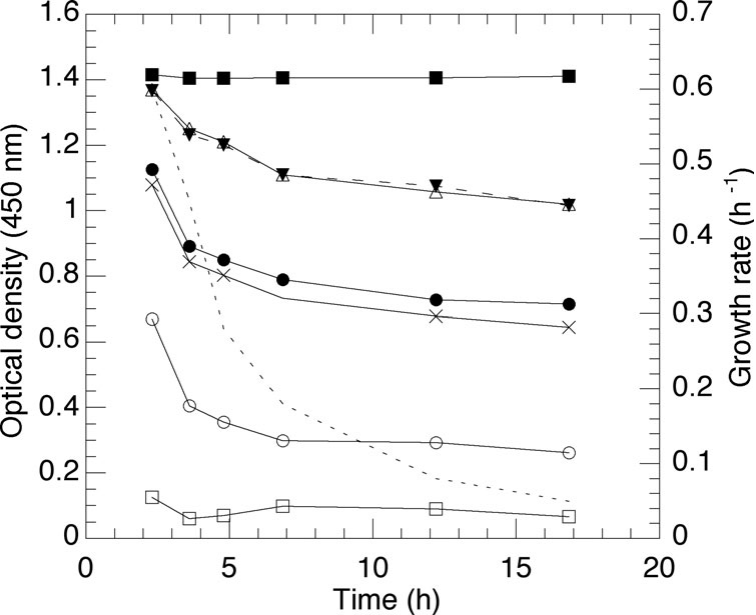
Effect of osmotic chock on *E.coli *W3110 cells represented by a drop in absorbation units at a specific addition. Addition from top to bottom: initial value, Tris-HCl, EDTA (100 mM), sucrose, lysozyme, water, EDTA (10 mM). The specific growth rate is indicated in the figure by a dashed line.

Osmotic shock is a frequently used method to release protein in the small scale. The methodology chosen as a model system gives a step-wise permeabilisation of the membrane, which is followed by breakage of the cell wall murein saccculus and formation of sphaeroplasts when lysozyme enters the cell [17]. Several conditions must be met before lysozyme can efficiently penetrate the outer membrane which was shown for several methods in the publication by Witholt et al [17]. The employed method relies six individual steps, as further described in material and methods. Figure 5 shows the drop in absorbance caused by each addition as a function of sampling at selected growth rates. At the top is shown the initial values and each following line in the diagram refers to the effect compared to the preceding addition. Consequently a large distance between two lines is a large effect and *vice versa*. The figure shows that the sucrose addition and the late addition of water results in the major effect on permeabilisation and, for unknown reason, also the initial dilution by Tris-HCl. The addition of lyzosyme, in itself, has no effect which is a result of the fact that it can only penetrate the membrane by the addition of water [17]. The effect, read as a change in absorbance is due to the change in the shape of the cells which was also clearly visible in the microscope. The growth rate dependent changes were evident between comparison of the first and second measuring points at high growth rate, specifically the result of the addition of water, but the effect stays thereafter more or less constant shown by the constant distance between the lines.

**Figure 6:**
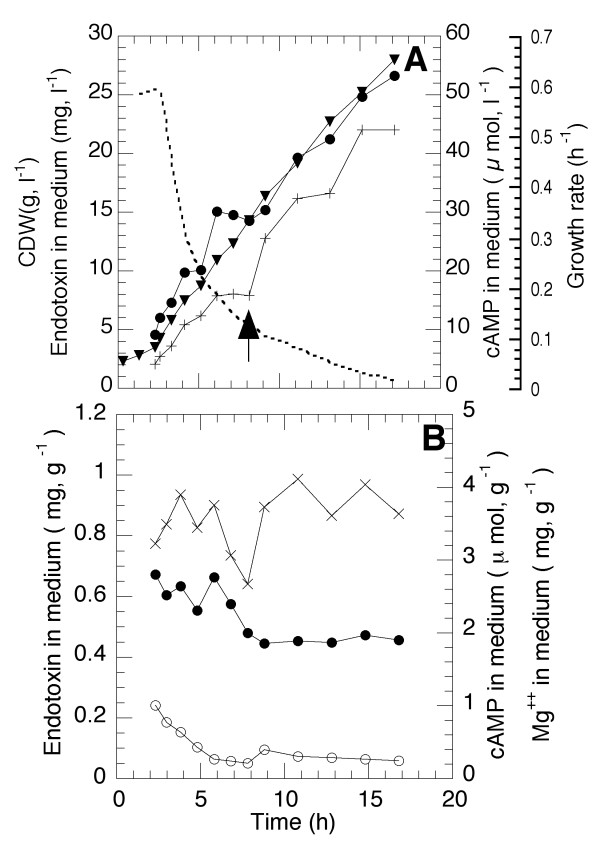
Accumulation of endotoxin (lipid A of lipopolysaccharide layer) and cAMP as a function of cultivation time. The specific growth rate is indicated in the figure by a dashed line. A. Volumetric accumulation of cAMP and endotoxin compared to CDW accumulation. The arrow indicates an addition of magnesium sulphate. B. Accumulation of cAMP and endotoxin in *μ*mol and mg per CDW, respectively.

The release of lipopolysaccharide, LPS, to the medium was measured by means of detection of the presence of a specific inner part of this structure, lipid A, which has properties of an endotoxin. This compound has to be removed in downstream processing of pharmaceutical proteins since it gives a toxic response in patients and must always be proven absent in the final product. It was considered likely that the changes in the underlying phospholipid and fatty acid structure might have large consequences for the release of this component to the medium which was to some extent confirmed by literature studies [12]. In Figure 6A the volumetric accumulation of endotoxin in the medium is seen as a function of time compared to cell mass accumulation. In this graph is also included the measurements of the cell metabolite, cyclic adenosine monophosphate (cAMP), which is important for carbon utilisation and which was proposed to constitute the signal for changes leading to membrane reorganisation during statis and stress [18]. The figure shows that all metabolites are accumulated with time.

In Figure 6B are plotted the specific accumulation of the parameters of figure 6A and it can be concluded that endotoxin and cAMP accumulation are constant and follows the cell mass accumulation.

**Figure 7:**
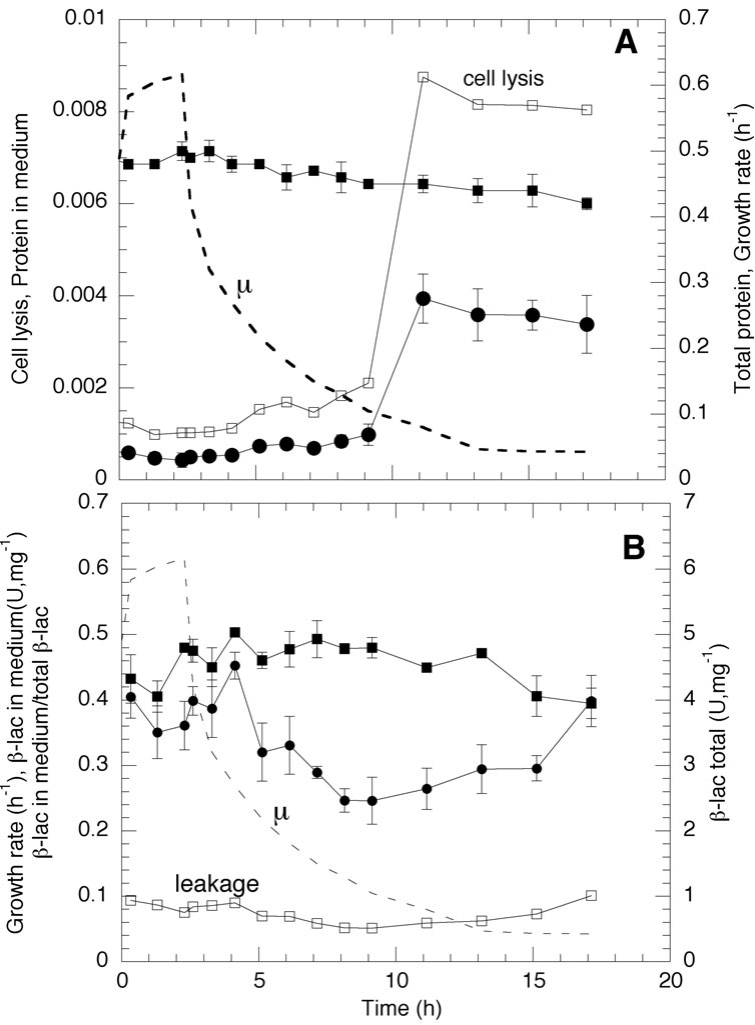
A. Total protein accumulation per CDW (filled squares), protein accumulation per CDW in the medium (circles) and cell lysis (protein in the medium/total protein, open squares) as a function of time. B. *β*-lactamase accumulation per CDW (filled squares), *β*-lactamase accumulation in the medium per CDW (circles) and leakage from periplasm to medium (*β*-lactamase in medium/total *β*-lactamase, open squares). The specific growth rate is indicated in the figure by a dashed line.

A major concern of this work was the effect of membrane structural changes on protein leakage from the periplasm to the medium. Since it is clear that a periplasmic protein can be present in the medium both by cell lysis and by true leakage from the periplasm, cell lysis was registered as a function of cultivation time. This is shown in Figure 7A. The leakage to the medium due to cell lysis was detected both by total protein analysis and by presence of DNA in the medium (the latter results not shown). Both variables showed the same pattern. At low growth rates there seems to be a marked change of the amount of total protein in the medium but it should be noted that the value is exceedingly low. The conclusion is that during the present fedbatch operation cell lysis is at all times very low and does not exceed 1 %. Earlier data from continuous cultivation [9] has shown that both total and specific protein synthesis can vary at different growth rates. To understand if this takes place also in fedbatch cultivation the total protein was measured at different growth rates. In Figure 7A is seen that total protein accumulation in each cell is constant down to a growth rate of 0.2 h^-1 ^where after it slowly declines from 0.50 to a final value of 0.42 g protein, g cells^-1^.

In Figure 7B is plotted the time course of the formation of the specific protein of interest i.e. periplamsic protein *β*-lactamase, which is constitutively produced from its own promoter. The total *β*-lactamase production is shown as well as the specific production values in the medium per gram of cells. It is clear that during fedbatch cultivation the total amount of this specific protein follows the total protein accumulation pattern and is constant independent if calculated per cell mass or per total cell protein except for the values at very low growth rate. The *β*-lactamase value per gram of cells in the medium is thus slightly higher during the first part of the cultivation down to *μ *= 0.3 h^-1 ^where after the value declines. At very low growth rate there is yet another period of increased specific accumulation. It is realised that this latter period of slowly increased accumulation in the medium coincides with an increased however small lysis in the end of the cultivation. Since the leakage is not increased during this period lysis seems most likely cause for the accumulation. The level of *β*-lactamase in the medium amounts to approximately 10% of the total *β*-lactamase production throughout the cultivation as seen from the quotient of *β*-lactamase in the medium in relation to the total value.

## Discussion

The conditions during fedbatch leads to a structure, which is approximately constant with respect to phospholipids and saturated fatty acids compared to growth rate. The changes in lipid components arrive from changes in unsaturated fatty acids where the increased values above the maximum at 0.3 h^-1 ^is formed in expense of cyclic fatty acids which are accumulating at low growth rate. Compared to literature data the amount of cardiolipin is approximately 70 % higher than from earlier observations [9].

The resulting effects on the membrane function are several but are generally and overall characterised by their stability as a function of growth rate. Cell lysis is low at all times i.e. the membrane is not easily breaking-up and this is due to the high content of cardiolipin at all times which is further emphasised by high levels of cyclic fatty acids specifically at growth rates below 0.3 h^-1^. The high content of cardiolipin is to some extent balanced by high level of unsaturated fatty acids, which compensates the need for flexibility, permeability and movement of compounds within the membrane which growth rate. The growth dependent accumulation and the generally high level of unsaturated fatty acids influence the results of sonication. The high levels above *μ *= 0.3 h^-1 ^thus supports a weaker membrane, in this respect, while the substitution of unsaturated to cyclic fatty acids reduces the effect at low growth rate. The transport of protein over the membrane is however only slightly higher at higher amounts of unsaturated fatty acids which is implicated by the larger effect of enzyme/water penetration during osmotic chock and the leakage of the model product, *β*-lactamase, at high growth rate. In total, *β*-lactamase is however always present in the medium at a level of approximately 10 % of the total production due to that the membrane flexibility caused by unsaturated fatty acid is balanced by the high and constant amount of cardiolipin.

There is a very slight increase in cell lysis at the end of cultivation. The reduced capacity of this cell for total and specific protein production at low growth rate, which might be due to a reduced amount of ribosomes [1], low supply of carbon and energy and the comparatively high maintenance demand, makes it likely that also the protein content in the membranes is reduced with increased susceptibility to lysis as a result. The changes at low growth rate often results in a change in the shape of the cells which are known to become smaller and more round [19] which might influence the performance. Flow cytometric studies could however not reveal any major changes of these cells during the time of the process (data not shown).

Continuous cultivation gives theoretically always the highest productivity due to the reduced shut-down periods for cleaning and refilling. However this technique is rarely used in industrial production of recombinant protein production due to problems of separation of growth and product formation. The pharmaceutical industry will furthermore need a validation protocol, which must be derived for this technique and where the concept of batch reproducibility needs some clarification. There is also a tendency to use techniques which are since long established and to which the personnel are educated. However, sometimes reasons are not well developed and a problem is the lack of data for a scientific choice of production technique.

In table 1 and 2 continuous cultivation and fedbatch data are summarised as a function of growth rate in a process based on the same cell, the same vector, the same medium and the same product. Table 1 shows selected values of membrane components and data has been collected as to represent either a relatively high (0.6 h^-1^) or a low (0.05 h-^1^) growth rate. The table furthermore indicate if curves, with respect to growth rate, show a trend which reveals the existence of maximum/minimum values of the specific compound. Table 2 shows the comparative effects on a selection of functional parameters at the same growth rates.

One of the most significant structural differences between the cultivation techniques is the high content of CL in fedbatch cells in expense of both PG and PE. The formation of large amounts of CL is an obvious cell strategy to save energy and carbon by the release of glycerol to further metabolisation. Since the only difference between the two cultivation techniques is the constant conditions in continuous cultivation and the dynamically changing ones in fedbatch the increased need for carbon/energy must lie in the rapidly changing growth environment.

In fedbatch there are also more unsaturated fatty acids accumulated, which develop in expense of saturated fatty acids, which are higher in continuous cultivation. The fatty acid level is also more or less constant in CL, which promotes stability. The conclusion is that cells in fedbatch cultivation interpret the environmental signals in a way that leads to a membrane structure with several features that generally only develop during nutrient exhaust in stationary phase. This is not the case for cells growth in continuous cultivation which develop distinct characteristics at each stable growth rate. This leads to a fedbatch membrane that is rigid compared to cells in continuous cultivation. The overall result is fewer changes in the membrane function due to growth rate. This is represented by constant leakage, constant lysis and an almost constant effect of permeabilisation. The fedbatch membrane shreds also less endotoxin although levels are low for both cultivation techniques. However, the fedbatch membrane is sensitive to sonication, specifically at high growth rate due to the comparatively high amount of unsaturated fatty acids and thus releases more *β*-lactamase due to the growth dependent changes of this fatty acid.

From the comparative data of table 1 and 2, from both process techniques it is evident that there is a growth dependent switch-point in the membrane regulation at approximately 0.3 h^-1^. However, this is not allowed to develop in fedbatch to the extent seen in continuous cultivation where a specific growth rate is kept much longer of the process time. The result is that no optimum in the leakage to the medium is clearly established in fedbatch cultivation and the fedbatch process cannot be designed for this since a constant growth rate of 0.3 h^-1 ^cannot be kept while at the same time leading to a high cell accumulation.

Protein transport is generally considered to benefit from a high content of PG and unsaturated fatty acids. This accumulation leads to high leakage which is verified by the continuous cultivation leakage optimum [9] and which leads to a comparatively high overall level in fedbatch (present data). The high fedbatch content of unsaturated fatty acids is however counteracted by signals leading to reduce levels of PG in favour of CL. The control of cellular activities at the switch-point leading to the establishment of the membrane compound pattern cannot be clarified at this point. However, it is clear that it is not the signals relating to the accumulation of cAMP. This compound is indeed a function of the growth rate, but not of the accumulation of the structural components.

The overall conclusion is thus that if leakage is undesired, fedbatch should not be used but the process could well be run in continuous cultivation at low growth rate. If an increased leakage is preferred this can be optimised in the continuous operation mode principally by change of growth rate but probably also by mutants accumulating a combination of phosphatidylglycerol and unsaturated fatty acids if these are stable enough for prolonged cultivation.

## Conclusion

The stability of the fedbatch membrane, which is not growth dependent, is the strength of this cultivation technique. The time of cell harvest is thus not critical since the membrane strength and function does not rapidly change which allows for flexibility in operation. The stability of the fedbatch derived cell membrane might thus also by the same reasoning be an advantage in further downstream processing however depending on the chosen unit operations.

The merits of the cell characteristics in continuous cultivation are due to the possibility to run the cultivation at a feed rate which is optimal for membrane leakage independent if this is to be small or maximised.

Total and specific protein production is lowered at low growth rate and the data points to that recombinant protein production should not be performed below a growth rate of 0.2 h^-1 ^and induction should thus take place well above this value. This is a considerable drawback in fedbatch cultivation, which relies on high cell density accumulation leading to low growth rates before induction but easily controlled in continuous cultivation. The coupling of the productivity and even product quality to a specific growth rate further strengthens the implication of using continuous cultivation.

The conclusion is that the drawbacks of industrial operation of continuous cultivation in the biopharmaceutical industry earlier mentioned should be preferably be solved to explore the benefits that are obviously present with this technique.

## Methods

### Strain and medium

*Escherichia coli *W3110 (F^-^, *λ*^-^, IN (rrnD-rrnE)) [20], a K12 derivative with a maximum growth rate, in the selected medium and temperature, of 0.65 h^-1^, was used. The plasmid pBR322 (Pharmacia Biotech, gene Bank Accession number V0119) was transferred to the host cell. This plasmid carries the genes *bla *and *tet *where *β*-lactamase (TEM-1) was used as the model product. The maximum levels were approximately 1 % of the total protein. The medium for the bioreactor and the feed was a mineral salts medium composed of (g, l^-1^): (NH_4_)_2_SO_2_, 7.0; KH_2_PO_4_, 1.6; Na_2_HPO_4 _*2H_2_O, 6.6; (NH_4_)_2_-H-citrate, 0.5. Glucose was autoclaved separately and added to bioreactor together with a sterile filtered trace element solution and 1.0 M MgSO_4 _(1 ml, l^-1^, respectively). A further addition of magnesium, of the same amount, was done at eight hours of feeding. The glucose concentration in the feed was 300 g, l^-1^. The trace elements solution was composed of (per litre): CaCl_2 _*2H_2_O, 0.5g; FeCl_3 _*6H_2_O, 16.7 g; ZnSO_4 _*7H_2_O, 0.18 g; CuSO_4 _*5H_2_O, 0.16g; MnSO_4 _*4H_2_O, 0.15 g; CoCl_2 _*6H_2_O, 0.18 g; Na-EDTA, 20.0 g.

### Cultivation Conditions

Four independent fedbatch cultivations were performed in the following manner: inoculum was prepared from a shake flask culture grown overnight at 37°C at 150 rpm. This was transferred into the bioreactor when an optical density at 600 nm (OD_600_) of 2–3 was reached from which point the fedbatch cultivation was performed. The experiments were carried out in a 14-l bioreactor with a working volume of 8.0 l. The bioreactor was equipped with an air sparger and the air-flow was 0.5–1.0 vvm. By varying of the stirrer speed up to 1000 rpm the dissolved oxygen concentration did never go below (30 ± 2) % air saturation. A pH of 7.0 was kept by addition of 28% (w/w) ammonia solution. Temperature was controlled at 37°C. The cultivations were started as batch cultures with an initial glucose concentration of 2 g, l^-1^. The feed was started when glucose was almost exhausted. The feed profile consists of two different phases where an exponential feed was followed by a constant feed phase. The exponential feed profile was calculated from the following equation:

F(t) = F_0 _* e^-*μ*t^

where F is the feed rate (L/h), *μ *the desired specific growth rate (1/h) and t is the process time (h). The calculation of the initial feed rate, F_0_, is based on a mass balance at the time of the feed i.e. after the batch growth assuming: pseudo steady state growth during the feed phase, a theoretical yield coefficient (Y_x/s_) of 0,5 g, g^-1 ^and a negligable amount of the limiting substrate in the reactor.

F_0 _=(μ *V*X)/(S_i_*Y_x/s_)

Where Y_x/s _is the cell yield on glucose (g/g), X is the cell concentration at F_0 _(g/L), V is the culture volume (L) and S_i _is the glucose concentration in the feed (g/L).

The continuous cultivation conditions and data, compared to in the conclusions section, were reported elsewhere [9]. These were designed for steady states at similar growth rates to those reported from the fedbtach cultivations of this paper. Steady state was kept for approximately eight generations and the same strains, vectors and media were used.

### Analyses

#### Cell mass, substrate and metabolites

The cell growth was followed by optical density at 600 nm. Biomass concentration was determined as cell dry weight (CDW, g, l^-1^) by 10 min centrifugation (4500 rpm, 2250 g) of 3*5 ml of cell suspension in preweighed test tubes and drying of the pellet overnight at 105°C before weighing. The supernatant from the CDW samples was sterile filtered and used for acetic acid and cyclic adenosine monophosphate, cAMP, analysis. Acetic acid was measured with the enzymatic method from Boehringer Mannheim GmbH (Cat. No 148 261) and cAMP was analysed by an enzyme immunoassay system from Amersham Pharmacia Biotech AB (code RPN 225).

Samples for glucose analysis were taken as described earlier [21]. The *β*-lactamase assay was performed as described in reference [22]. Inductively Coupled Plasma (ICP) analysis was used for magnesium determination. Total protein and DNA analysis were used for cell lysis detection. Total-protein was measured according to the method by [23] using bovine serum albumin as standard (Sigma Chemical Co, USA). DNA was measured by a fluorescence assay and quantified with Hoechst 33258 (Sigma Chemical Co, USA) according to the Dyna Quant 200 application method (Pharmacia, Sweden). The amounts of endotoxin i.e. lipid A, the inner part of the lipopolysaccharide (LPS) structure, was measured by the chromogenic Limulus method (Chromogenix, LAL, U.S. License No. 1197).

#### Mechanical strength of the cell membrane

The mechanical strength was estimated by sonication (Vibra-Cell, Sonics & Materials, USA) at constant frequency, cell dry mass, sample volume and acoustic power. Samples were placed in a water bath at +4°C during cell disruption to prevent overheating. The release of *β*-lactamase protein was determined at the time events of 0.5, 1.0, 1.5 and 2.0 minutes and compared to cell that was disintegrated in a high-pressure homogeniser (French press FA-073) at 55 bar. Data from French press was set as the 100% disruption level and all other values were taken as a percentage of this value.

#### Chemical strength of the cell membrane

Chemical strength was estimated by permeabilisation as described by Witholt et al [17]. The following steps were thus undertaken: (1) t = 0 min, 0.5 g, l^-1 ^cultures were harvested and suspended in 0.3 ml, 200 mM Tris-HCL (pH 8.0), (2) t = 1 min, 0.5 *μ*l, 100 mM EDTA (pH 7.6) was added, (3) t = 2 min, 0.5 ml, 1 M sucrose (pH 8.0) was added thus giving excess of EDTA over Mg^2+ ^ions, (4) t = 3.5 min, egg white lysozyme (EC 3.2.1.17, C. F. Boehringer und Soehne GmbH, Mannheim, Germany) was added to a final concentration of 60 *μ*g/ml, (5) t = 4 min, the cell suspension was exposed to a mild osmotic shock by two-fold dilution in water to trigger lysozyme penetration of the outer membrane, and finally (6) t = 8 min, the cell suspension was diluted 11-fold in 10 mM EDTA (pH 7.6). Permeabilisation was recorded by optical density at 450 nm. The samples taken at different growth rate were diluted to the same cell concentration (0.5 g, l^-1^) and the same volume (0.2 ml) was used at the sampling point.

#### Phospholipids and fatty acids analysis

Phospholipids and fatty acids were determined by the method of Arneborg [24] where the total and individual phospholipid content was quantified by an inorganic phosphorous assay after extraction and thin liquid chromatography. The presented value is the mol% of individual to the total phospholipids. The standard deviation was +/- 0.03 %. Fatty acids were released from extracted phospholipids by transmethylation with KOH in methanol. The methyl esters are thus representing the actual fatty acid. Chromatography was performed with C13:0 as an internal standard as described by [25]. The presented value is the mol% of individual to the total fatty acids (shortened for the number of carbon atoms according to: C14:0, C16:0, C16:1, C18:1 and 17:cyc). The methodology is further described in reference [9].

## Author's contributions

AS made the fermentations, analyses and data treatments and evaluation. The project was conceived by GL who further participated in the project planning, design and data evaluation. All authors read and approved the final manuscript.

## Figures and Tables

**Table 1:** Membrane structural changes as a function of growth rate and cultivation technique

***STRUCTURE***^1^	***Continuous cultivation***			***Fed-batch cultivation***			***Comment***
***Parameter***	*Low μ*	*Max/Min*	*High μ*	*Low μ*	*Max/Min*	*High μ*	Min/Max values which correlate with *μ *= 0.3 h^-1^

Phospholipids (%)							
PE	70	75	78	77	n.p.	77	
PG	16	18	15	12	**	11	Max value only in CC
CL	8	7	7	12	*	12	
PS	7	-	-	-	-	-	Compound not produced in FB
Fatty acids (%)							
C14:0	3	1	2	3	n.p.	3	Min value only in CC
C16:0	53	49	52	44	*	44	Min value only in CC
C16:1	16	22	21	19	28	26	Max value only in CC
C18:1	8	20	14	12	21	18	Max value only in CC
C17:1	20	7	11	22	9	9	Min value only in CC

^1^Summary of the structural differences in continuous and fed-batch cultivation as a function of growth rate. Results compared to data from Shokri et al 2002. N.p.: max/min not present. CC: continuous cultivation, FB: fed-batch cultivation. * value is slightly decreased compared to the value at high μ. ** value is slightly increased compared to the values at high μ. A high growth rate refers to μ = 0.6 h^-1 ^and a low growth rate to μ = 0.05 h^-1^.

**Table 2:** Membrane functional changes as a function of growth rate and cultivation technique

***FUNCTION***^2^	***Continuous cultivation***			***Fed-batch cultivation***			***Comment***
***Parameter***	*Low μ*	*Min/Max*	*High μ*	*Low μ*	*Min/Max*	*High μ*	

Total protein production (per CDW)	R	High	High	R	High	High	Yield drops from the theoretical 0.5 to approx. 0.4
Specific protein production (per tot prod.)	R	High	High.	R	High	High	Model substance: periplasmic *β*-lactamase
Cell lysis (%)	8	1	1	1	<1	<1	
Effect of sonication (% release of specific protein)	56	78	73	57	80	85	Result of 1 min sonication
Endotoxin release (% of CDW)	1	1	1	<1	<1	<1	
Periplasmic leakage	1.5	19	7	10	10	9	

*^2^Summary of the structural differences in continuous and fed-batch cultivation as a function of growth rate. Results compared to data from Shokri et al 2002. A high growth rate refers to μ = 0.6 h^-1 ^and a low growth rate to μ = 0.05 h^-1^*.
